# Association and interaction effect of UCP2 gene polymorphisms and dietary factors with congenital heart diseases in Chinese Han population

**DOI:** 10.1038/s41598-021-88057-2

**Published:** 2021-04-22

**Authors:** Senmao Zhang, Xiaoying Liu, Tingting Wang, Lizhang Chen, Tubao Yang, Peng Huang, Jiabi Qin

**Affiliations:** 1 Department of Epidemiology and Health Statistics, Xiangya School of Public Health, Central South University, 110 Xiangya Road, Changsha, 410078 Hunan China; 2 Hunan Provincial Key Laboratory of Clinical Epidemiology, Changsha, Hunan China; 3 Department of Cardiothoracic Surgery, Hunan Children’s Hospital, 86 Ziyuan Road, Hunan 410007, Changsha, China; 4 NHC Key Laboratory of Birth Defect for Research and Prevention, Hunan Provincial Maternal and Child Health Care Hospital, Changsha, Hunan China; 5 Guangdong Cardiovascular Institute, Guangdong Provincial People’s Hospital, Guangdong Academy of Medical Sciences, Guangzhou, Guangdong China

**Keywords:** Genetics, Cardiology, Risk factors

## Abstract

Congenital heart diseases (CHDs) are the most common birth defects and the leading cause of non-infectious deaths in infants, with an unknown etiology. We aimed to assess the association of genetic variations in UCP2 gene, dietary factors, and their interactions with the risk of CHDs in offspring. The hospital-based case–control study included 464 mothers of children with CHDs and 504 mothers of healthy children. The exposures of interest were maternal dietary factors in early pregnancy and UCP2 genetic variants. Logistic regression analyses were used to assess the association and interaction of UCP2 gene and dietary factors with CHDs. Our results found that the polymorphisms of UCP2 gene at rs659366 and rs660339, together with maternal dietary factors including excessive intake of pickled vegetables and smoked foods were associated with increased risks of CHDs in offspring. Regular intake of fresh meat, fish and shrimp, and milk products were associated with lower risks of CHDs in offspring. Besides, positive interaction between the dominant model of rs659366 and excessive intake of pickled vegetables was found in the additive interaction model (RERI = 1.19, *P* = 0.044). These findings provide the theoretical basis for gene screening and a new clue for the prevention of CHDs in offspring.

## Introduction

Congenital heart diseases (CHDs) were the most common birth defects in the world, accounting for one-third of congenital anomalies, with an estimated prevalence of 8.22 per 1000 live birth worldwide and 8.98 per 1000 live birth in China^1–3^. It has been estimated that approximately 1.35 million infants with CHDs are born each year globally, with one-third of them requiring surgical intervention in the first year of life, which causes heavy financial burden to families and society^2,4,5^. Although the development of surgical techniques has changed the natural history of CHDs dramatically and decreased the mortality of CHDs^6^, these patients with CHDs still have high risks for cardiovascular disease and neurodevelopmental disabilities in later life^7,8^.

Until now, although the etiology of CHDs is still unclear^5,6^, significant advancement has been achieved in the better understanding of the genetic etiology of CHDs in recent years. Mutations in NKX2.5, GATA4, TBX5, and ZIC3 genes have been repeatedly confirmed to be associated with CHDs^5^. The introduction of single-nucleotide polymorphisms (SNPs) array technology in the 1990s enabled genome-wide association studies (GWASs) in the late 2000s^9,10^. To date, GWAS and candidate gene strategies have identified lots of susceptibility genes of CHDs^5^. However, so far, the genetic variations observed are insufficient to fully explain the genetic contribution of CHDs. Ongoing research identifying novel genetic variations may provide new clues for the prevention of CHDs.

The uncoupling protein 2 (UCP2), encoded by the UCP2 gene, is a member of the mitochondrial inner membrane carrier family. UCP2 plays an essential role in regulating energy metabolism, insulin secretion, reactive oxygen species (ROS) production, as well as different cellular processes, including cell metabolism, cell proliferation and cell death^11,12^. Strong evidence suggests that the UCP2 gene polymorphisms may affect the expression/activity levels of UCP2 and subsequently affect related biological processes, which finally result in a variety of diseases, including metabolic diseases and cancer^12^. Until now, some SNPs of the UCP2 gene have been identified to be associated with cardiovascular disease^13^, diabetes^14^, obesity^15^, and cancer^16^. However, evidence regarding the association between UCP2 genetic variants and the risk of CHDs is very scarce.

Given that the above-mentioned diabetes and obesity have been recognized as risk factors of CHDs, it is indirectly suggested that SNPs of UCP2 gene may be related to the development of CHDs^17,18^. Meanwhile, the UCP2 gene is thought to be negative regulators of reactive oxygen species (ROS) generation^19,20^. Previous studies have demonstrated that the mitochondrial overproduction of ROS plays a crucial role in the development of the embryonic heart^21,22^, which directly suggests that SNPs of UCP2 gene may eventually lead to CHDs by regulating ROS generation. Therefore, we speculated that UCP2 gene polymorphisms may be associated with the risk of CHDs. Besides, it is generally believed that more than 85% of CHDs result from complex interactions between genetic variants and environmental factors^23^. Dietary factors are considered as a modifiable environmental factor, which is also associated with the risk of CHDs^24–27^. Furthermore, some studies have found a significant interaction between UCP2 gene and dietary intake on the development of metabolic diseases^28,29^. However, most published studies have focused on specific dietary nutrients or harmful substances in foods, but few studies explored the association between dietary factors and the risk of CHDs based on food categories. Thus, it is necessary to further investigate the association of maternal dietary factors based on food categories with the risk of CHDs in offspring.

Based on the above-mentioned factors, we build up a hypothesis that there are interaction effects between maternal dietary factors and UCP2 genetic variants jointly leading to CHDs. In this hospital-based case–control study, we examine the association and interaction effect of UCP2 gene polymorphisms and dietary factors with the risk of CHDs in offspring.

## Results

### Basic characteristics in case and control groups

The basic characteristics of 504 controls and 464 cases are shown in Table 1. The mean gestational age was 28.56 ± 4.70 in the case group, which was significantly higher than that in the control group 27.77 ± 5.40 (*P* = 0.016). In addition, we found that maternal education level, average annual income, history of diabetes, smoking history, second-hand smoke exposure history, history of drinking alcohol, and folic acid supplement in the case group were significantly different from the control group (*P* < 0.05). These factors which were significantly different across groups were adjusted when estimating the association of UCP2 gene polymorphisms and maternal dietary factors with the risk of CHDs.

**Table 1:** Baseline characteristics in the case and control groups.

Baseline characteristics	Control(n = 504)		CHDs (n = 464)		t/χ^2^	*P*
	N	%	N	%		
Gestational age (years)	28.56 ± 4.70		27.77 ± 5.40		2.399	0.016
**Education level**					12.306	< 0.001
Less than primary	6	1.2	66	14.2		
Junior high school	100	19.8	190	40.9		
Senior middle school	168	33.3	130	28.1		
College or higher	230	45.7	78	16.8		
**Average annual income (RMB)**				15.946	< 0.001	
≤ 50,000	144	28.6	372	80.2		
60,000–100,000	216	42.9	68	14.7		
110,000–150,000	46	9.1	10	2.1		
≥ 160,000	98	19.4	14	3.0		
**Body mass index**					2.446	0.294
< 18.5	126	25.0	98	21.2		
18.5–23.99	288	57.1	286	61.6		
≥ 24	90	17.9	80	17.2		
**History of diabetes**					28.414	< 0.001
No	482	95.6	398	85.8		
Yes	22	4.4	66	14.2		
**Smoking history**					14.046	< 0.001
No	494	98.0	432	93.1		
Yes	10	2.0	32	6.9		
**Second-hand smoke exposure history**					21.589	< 0.001
No	316	62.7	222	47.8		
Yes	188	37.3	242	52.2		
**History of drinking alcohol**					9.060	0.003
No	468	92.9	404	87.1		
Yes	36	7.1	60	12.9		
**Folic acid supplement**					23.917	< 0.001
No	34	6.7	78	16.8		
Yes	470	93.3	386	83.2		

*CHDs* congenital heart diseases.

### Association of UCP2 genetic variants and maternal dietary factors with the risk of CHDs

**Figure 1:**
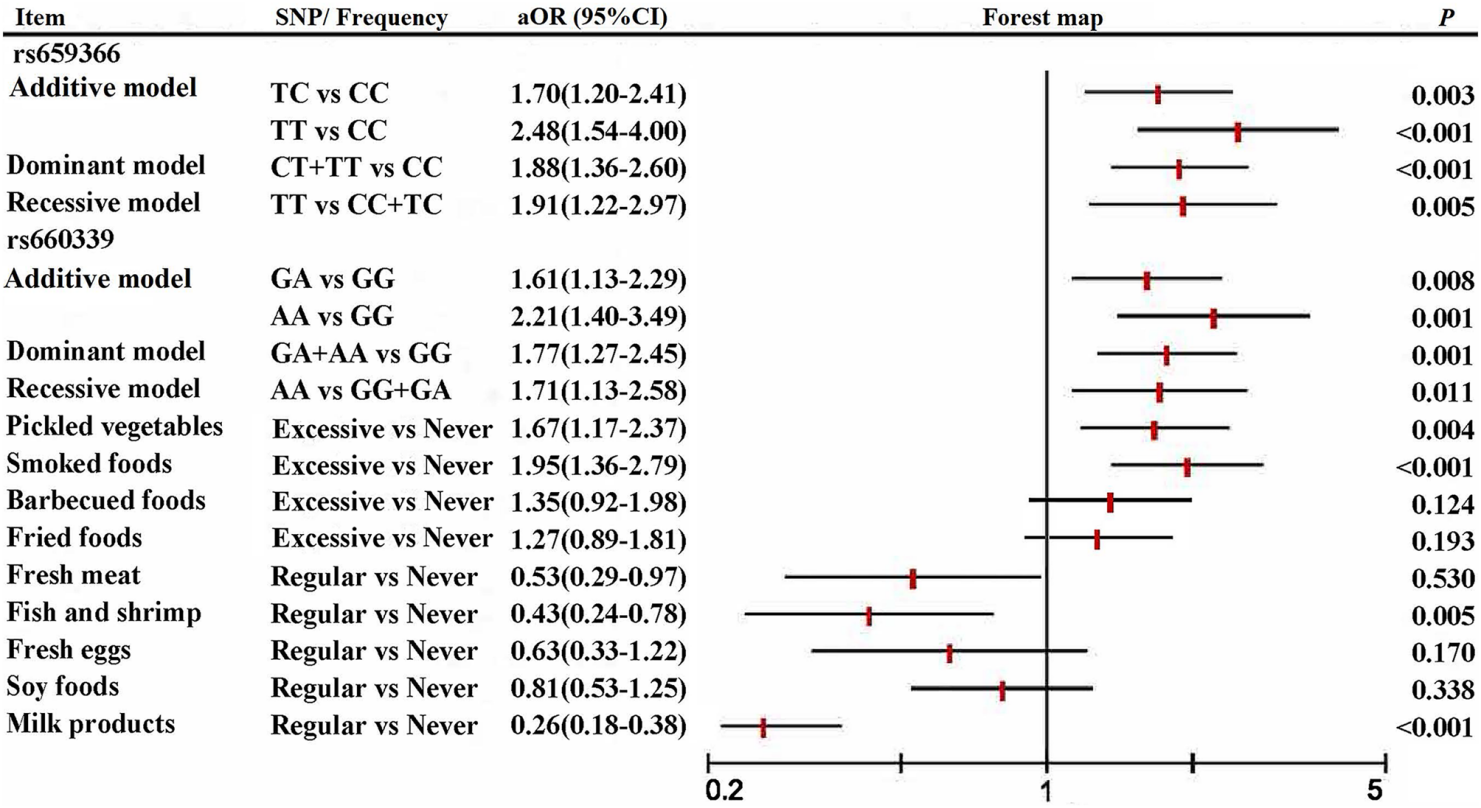
The association of the UCP2 gene and dietary factors with CHDs in multivariate analyses.

All SNPs of the UCP2 gene in the controls were conformed to Hardy–Weinberg equilibrium shown in Supplement Table S1 (*P* > 0.05). Meanwhile, the associations of UCP2 gene polymorphisms and maternal dietary factors with the risk of CHDs based on univariate analysis are shown in Table 2. After adjusting for basic characteristics that were significantly different across groups, the associations of UCP2 gene polymorphisms and maternal dietary factors with the risk of developing CHDs are examined under each gene model based on multivariate logistic regression (Fig. 1).

**Table 2:** The association of the UCP2 gene and dietary factors with CHDs in univariate analyses.

SNP	Controls	CHDs	cOR(95%CI)	*P*	Dietary factors	Controls	CHDs	cOR(95%CI)	*P*
	N(%)	N(%)				N(%)	N(%)		
**rs659366**					Pickled vegetables				
Additive model					Never	330(65.5)	253(54.5)	1.00	
CC	252(50.0)	158(34.0)	1		Excessive	174(34.5)	211(45.5)	1.58(1.22–2.05)	0.001
TC	192(38.1)	210(45.3)	1.74(1.32–2.31)	< 0.001	Smoked foods				
TT	60(11.9)	96(20.7)	2.55(1.75–3.73)	< 0.001	Never	276(54.8)	190(40.9)	1.00	
Recessive model					Excessive	228 (45.2)	274(59.1)	1.75(1.35–2.25)	< 0.001
CC + TC	444(88.1)	368(79.3)	1		Barbecued foods				
TT	60(11.9)	96(20.7)	1.93(1.36–2.74)	< 0.001	Never	376(74.6)	293(63.1)	1.00	
Dominant model					Excessive	128(25.4)	171(36.9)	1.71(1.30–2.26)	< 0.001
CC	252(50.0)	158(34.1)	1		Fried foods				
TC + TT	252(50.0)	306(65.9)	1.94(1.49–2.51)	< 0.001	Never	306(60.7)	235(50.6)	1.00	
**rs660339**					Excessive	198(39.3)	229(49.4)	1.51(1.17–1.94)	0.002
Additive model					Fresh meat				
GG	218(43.3)	136(29.3)	1		Never	22(4.4)	66(14.2)	1.00	
GA	212(42.0)	216(46.6)	1.63(1.23–2.17)	0.001	Regular	482(95.6)	398(85.8)	0.26(0.17–0.45)	< 0.001
AA	74(14.7)	112(24.1)	2.43(1.69–3.49)	< 0.001	Fish and shrimp				
Recessive model					Never	22(4.4)	98(21.1)	1.00	
GG + GA	430(85.3)	352(75.9)	1		Regular	482(95.6)	366(78.9)	0.17(0.11–0.28)	< 0.001
AA	74(14.7)	112(24.1)	1.85(1.34–2.56)	< 0.001	Fresh eggs				
Dominant model					Never	24(4.8)	60(12.9)	1.00	
GG	218(43.3)	136(29.3)	1		Regular	480(95.2)	404(87.1)	0.34(0.21–0.55)	< 0.001
GA + AA	286(56.7)	328(70.7)	1.84(1.41–2.40)	< 0.001	Fresh vegetables				
**rs591758**					Never	2(0.4)	2(0.4)	1.00	
Additive model					Regular	502(99.6)	462(99.6)	0.92(0.13–6.56)	0.934
GG	180(35.7)	160(34.5)	1		Soy foods				
GC	228(45.2)	212(45.7)	1.05(0.79–1.39)	0.756	Never	70(13.9)	144(31.0)	1.00	
CC	96(19.1)	92(19.8)	1.08(0.76–1.54)	0.679	Regular	434(86.1)	320(69.0)	0.36(0.26–0.49)	< 0.001
Recessive model					Milk products				
GG + GC	408(81.0)	372(80.2)	1		Never	82(16.3)	241(51.9)	1.00	
CC	96(19.0)	92(19.8)	1.05(0.76–1.45)	0.759	Regular	422(83.7)	223(48.1)	0.18(0.13–0.24)	< 0.001
Dominant model									
GG	180(35.7)	160(34.5)	1						
GC + CC	324(64.3)	304(65.5)	1.06(0.81–1.38)	0.688					

*CHDs* congenital heart diseases; *cOR* crude odds ratio; *CI* confidence interval; *SNPs* single nucleotide polymorphisms;

*Recessive model means homozygous variant vs heterozygous variant + homozygous wild-type.

^†^Dominant model means homozygous variant + heterozygous variant vs homozygous wild-type.

Overall, our study showed that the polymorphisms of UCP2 gene at rs659366 and rs660339 were significantly associated with the risk of CHDs in offspring. For rs659366, mother carrying the TC genotype (aOR = 1.70, 95% CI 1.20–2.41, *P* = 0.003) or TT genotype (aOR = 2.48, 95% CI 1.54–4.00, *P* < 0.001) compared with the TT genotypes were more likely to have the risk of developing CHDs. Additionally, the dominant model (aOR = 1.88, 95% CI 1.36–2.60, *P* < 0.001) and recessive model (aOR = 1.91, 95% CI 1.22–2.97, *P* = 0.005) of rs659366 also were significantly associated with increased risks of CHDs in offspring. For rs660339, mothers carrying the GA genotype (aOR = 1.61, 95% CI 1.13–2.29, *P* = 0.008) or AA genotype (aOR = 2.21, 95% CI 1.40–3.49, *P* = 0.001) were significantly higher risks of CHDs compared with the GG genotypes. Meanwhile, the dominant model (aOR = 1.77, 95% CI 1.27–2.45, *P* = 0.001) and recessive model (aOR = 1.71, 95% CI 1.13–2.58, *P* = 0.011) of rs660339 also were significantly associated with increased risks of CHDs in offspring.

Besides, our results showed that mothers with excessive intake of pickled vegetables (aOR = 1.67, 95% CI 1.17–2.37, *P* = 0.004) and smoked foods (aOR = 1.95, 95% CI 1.36–2.79, *P* < 0.001) were more likely to increase the risk of developing CHDs compared to those with never intake. In contrary, regular intake of fresh meat (aOR = 0.53, 95% CI 0.29–0.97, *P* = 0.038), fish and shrimp (aOR = 0.43, 95% CI 0.24–0.78, *P* = 0.005), and milk products (aOR = 0.26, 95% CI 0.18–0.38, *P* < 0.001) were lower risk of developing CHDs than in those who did not consume.

### Interaction of UCP2 gene and maternal dietary factors with the risk of CHDs

Table 3 shows the effect of the interaction of the dominant model of rs659366 and maternal dietary factors on the risk of CHDs. After adjusting for basic characteristics that were significantly different across groups, our results suggested that there was statistically significant positive interaction between the dominant model of rs659366 and excessive pickled vegetables (RERI = 1.19, 95%CI 0.03–2.35, *P* = 0.044). Compared with mother carrying the CC genotype and having no pickles vegetable intake, those carrying the CT + TT genotype with excessive pickled vegetables intake have a higher risk of CHDs (OR = 2.96, 95% CI 1.90–4.59, *P* < 0.001). Besides, we further analyzed the independent effect of maternal UCP2 genetic polymorphisms on the risk of CHDs by stratification of maternal pickled vegetables intake. Our results suggested that the risk of CHDs in offspring was significantly increased among mothers carrying the CT + TT genotype of rs659366 with excessive pickled vegetables intake compared with those carrying the CT + TT genotype of rs659366 with never pickled vegetables intake (aOR: 2.04, 95% CI 1.34–3.12, *P* = 0.001).

**Table 3:** Interaction between the dominant model of rs659366 and maternal dietary factors for the risk of CHDs.

Dietary factors	CC		TC + TT		aOR(95%CI) for genotypes within strata of dietary factors	RERI(95%CI)
	Case/control	aOR(95%CI)	Case/control	aOR(95%CI)		
**Pickled vegetables**						**1.19(0.03–2.35)** ***P = 0.044***
Never	99/162	1(Ref.)	154/168	1.45(0.96–2.17) *P* = 0.074	1.45(0.96–2.17) *P* = 0.074	
Excessive	59/90	1.32(0.80–2.18) *P* = 0.278	152/84	2.96(1.90–4.59) *P* < 0.001	2.24 (1.33–3.76) *P* = 0.002	
aORs (95%CI) for excessive pickled vegetables intake within strata of genotype		1.32(0.80–2.18) *P* = 0.278		2.04 (1.34–3.12) *P* = 0.001		
**Smoked foods**						0.14(-1.62–1.91) *P* = 0.873
Never	57/144	1(Ref.)	133/132	2.27(1.43–3.60) *P* < 0.001	2.27(1.43–3.60) *P* < 0.001	
Excessive	101/108	2.94(1.80–4.81) *P* < 0.001	173/120	4.36(2.71–7.00) *P* < 0.001	1.92 (1.26–2.92) *P* = 0.020	
aORs (95%CI) for excessive smoked foods intake within strata of genotype		2.94(1.80–4.81) *P* < 0.001		1.48 (0.95–2.31) *P* = 0.085		
**Fresh meat**						0.04 (-1.75–1.83) *P* = 0.965
Regular	130/241	1(Ref.)	268/241	1.82 (1.29–2.56) *P* = 0.001	1.82 (1.29–2.56) *P* = 0.001	
Never	11/28	1.60 (0.80–3.22) *P* = 0.186	11/38	2.46 (1.33–4.56) *P* = 0.004	1.53 (0.64–3.64) *P* = 0.329	
aORs (95%CI) for never fresh meat intake within strata of genotype		1.60 (0.80–3.22) *P* = 0.186		1.36 (0.74–2.46) *P* = 0.321		
**Fish and shrimp**						-2.41(-8.11–3.28) *P* = 0.406
Regular	118/242	1(Ref.)	248/240	1.99 (1.42–2.80) *P* < 0.001	1.99 (1.42–2.80) *P* < 0.001	
Never	40/10	5.88 (2.52–13.73) *P* < 0.001	58/12	4.45 (2.18–9.10) *P* < 0.001	0.76 (0.26–2.61) *P* = 0.605	
aORs (95%CI) for never fish and shrimp intake within strata of genotype		5.88 (2.52–13.73) *P* < 0.001		2.24 (1.10–4.52) *P* = 0.025		
**Milk products**						2.08 (-3.98–8.13) *P* = 0.502
Regular	73/211	1(Ref.)	150/211	1.93 (1.13–3.28) *P* = 0.016	1.93 (1.13–3.28) *P* = 0.016	
Never	85/41	4.47 (3.02–6.62) *P* < 0.001	156/41	7.47 (3.32–16.82) *P* < 0.001	1.64 (0.91–2.98) *P* = 0.106	
aORs (95%CI) for never milk products intake within strata of genotype		4.47 (3.02–6.62) *P* < 0.001		4.20 (2.60–6.80) *P* < 0.001		

*CHDs* congenital heart diseases; *aORs* adjusted odds ratios; *95%CI* 95% confidence intervals; *RERI* The relative excess risk due to interaction. Adjusted for baseline characteristics that were significantly different among two groups.

Supplement Tables S2–S4 separately shows the effect of the interaction of the recessive model of rs659366, the dominant model of rs660339, and the recessive model of rs660339 and maternal dietary factors on the risk of CHDs. After adjusting for basic characteristics that were significantly different across groups, the interaction between the recessive model of rs659366, the dominant model of rs660339, and the recessive model of rs660339 and maternal dietary factors were not found in the additive interaction model (all *P* values of RFRI > 0.05).

### Linkage disequilibrium (LD) test and haplotype analysis

The r-square values of the linkage disequilibrium test among SNPs of maternal UCP2 gene are summarized in Table 4. Our results showed that there were not strong correlations (all r^2^ < 0.8) among these three SNPs of UCP2 gene (r^2^ = 0.630 between rs659366 and rs660339; r^2^ = 0.588 between rs659366 and rs591758; r^2^ = 0.623 between rs660339 and rs591758). Linkage disequilibrium (LD) analysis of the UCP2 SNPs between cases and controls is shown in Supplement Fig. S1. The r-square values and log-odds scores indicated that there were no potential linkage disequilibrium blocks between these SNPs. Therefore, we did not perform a haplotype analysis of these SNPs.

**Table 4:** Degree of linkage disequilibrium of the UCP2 SNPs between cases and controls.

r^2^	rs659366	rs660339	rs591758
rs659366	1		
rs660339	0.630	1	
rs591758	0.588	0.623	1

## Discussion

CHDs are the most common birth defects in the world and the major cause of infant non-infection mortality^30^. Although most studies have found that genetic and environmental factors were closely associated with CHDs, the etiology of most cases remains unknown^5,6^. In the present study, we first assess the association of the the polymorphisms of UCP2 gene and the risk of CHDs. Our results suggested that the polymorphisms of UCP2 gene at rs659366 and rs660339 were associated with increased risks of CHDs after adjustment for confounding factors, which indicated that UCP2 gene could play an important role in the development of CHDs. Over the past decades, there were no studies for whether the UCP2 gene could affect the risk of CHDs in offspring. However, some evidence has suggested that the mutations of UCP2 genes can affect the activity or expression levels of UCP2 by increasing the coupling of oxidative phosphorylation, which might cause the reduction of energy expenditure and subsequently contribute to the development of obesity and diabetes^14^^.^ At present, most previous studies have found that two common polymorphisms of UCP2 gene including rs659366 (located in the promoter region) and rs660339 (a missense variant in exon 4) were closely associated with obesity and diabetes^12,31,32^. Furthermore, maternal obesity and diabetes have been identified as risk factors of CHDs, which indirectly indicated that genetic variants of UCP2 may be associated with the risk of CHDs in offspring.

Besides, the polymorphisms of UCP2 gene also are associated with the excessive accumulation of ROS^33,34^. Previous studies have suggested that excessive accumulation of ROS may lead to the abnormal development of the embryonic heart through irreversible damage to cell membranes, DNA, and other cellular structures^13,21,35^. Meanwhile, excessive accumulation of ROS also may exert cytotoxic effects in cardiomyocytes, thereby resulting in the abnormal development of the embryonic heart^21,36^. In a word, findings from the present study support our results. This implies that UCP2 gene might become the susceptibility gene for CHDs in offspring, and helps to provide a new thread for finding candidate genes for CHD in the future.

In the present study, we also analyzed the association of maternal dietary factors with the risk of developing CHDs in offspring. Our results suggested that excessive intake of pickled vegetables and smoked food were significantly associated with increased risks of CHDs in offspring. Pickled vegetables contain large amounts of nitrite. The previous study has strongly indicated that excessive intake of nitrite can directly affect atrioventricular valve formation by yielding too much NO signaling, thereby causing abnormal development of zebrafish heart^37^. Besides, smoked food usually contained a high concentration of polycyclic aromatic hydrocarbons (PAH). After ingestion, PAH compounds can be transferred through the placenta to the fetus^38^. Their metabolites may bind to DNA to form PAH-DNA adducts, which might exert reproductive and developmental toxicity effects^39^. Previous animal studies and population research^40–43^ have found that the exposure of PAH during embryonic life may derail the concerted expression of genes critical to normal cardiovascular system development and increase the risk of developing CHDs in offspring. On the contrary, our results showed that the regular intake of fresh meat, fish and shrimp, and milk products may decrease the risk of developing CHDs in offspring, which is consistent with most previous studies^44–46^. These three foods contain large amounts of proteins, lipids, unsaturated fatty acids, essential amino acids, and minerals, which are beneficial to the heart development of the embryonic.

Besides, we also detected the interactions between the UCP2 gene and maternal dietary factors on the risk of developing CHDs in offspring. Our study firstly reported a positive interaction between pickled vegetable intake and the dominant model of rs659366. Our results suggested that the estimated joint effect on the additive model of rs659366 and excessive intake of pickled vegetables together was greater than the sum of the estimated effects of genotype alone and pickled vegetable intake alone, which indicated that genetic and dietary factors jointly cause the occurrence of CHDs in offspring. Although previous studies^28,29^ have found a significant interaction between the UCP2 gene and dietary factors on the development of metabolic diseases, the involved pathways and the regulatory mechanism are still unclear and need further research. The observed interaction provides a new idea for further finding the etiology of CHDs in offspring and providing prevention strategies.

This study had some limitations. Firstly, because many complicated severe CHDs during pregnancy may have been induced by therapeutic induction or the fetus has died in utero, we only selected CHDs treated with surgery after birth as the study participant. It was impossible to select samples by random sampling, which inevitably led to selection bias and affected the representativeness of the results to a certain extent. Secondly, maternal diet during pregnancy was retrospectively reported by the mother with CHDs waiting for surgery in the hospital, therefore, recall bias was inevitable. Although a previous study^47^ has suggested that maternal diet during pregnancy could be recalled well after birth, we cannot overlook the possible limitation of recall bias. Thirdly, although some previous studies^28,29^ have found a significant interaction between the UCP2 gene and dietary intake on the development of metabolic diseases, the involved pathways and the regulatory mechanism of interaction between genetic and dietary factors is unclear, which emphasizes the necessity of further research. Fourth, the specific inclusion and exclusion criteria, as well as a very local population in our study may limit the extrapolation of our findings and make it difficult to compare directly with other studies. Fifth, the limitation that only three SNPs of UCP2 gene were selected in our study cannot be ignored, because UCP2 gene has more than ten SNPs. Although our study suggested the significant statistical association between UCP2 gene and the risk of CHDs in offspring, more SNPs should be detected in the future to obtain more genetic information and provide more clues for the study of genetic susceptibility sites of CHDs. Sixth, residual confounders are always of concern in observational studies. Although the present study adjusted for a wide range of potential confounders for CHDs, we still could not exclude the possibility that other unmeasured or inadequately measured factors have confounded the true association. Besides, due to the sample size is still relatively small, we only focus on the risk of total CHDs when assessing the effect of maternal dietary factors, the polymorphisms of UCP2 gene, and their interactions on CHDs in offspring. These limitations highlight the urgent need for large samples, multicenter, prospective, and different ethnic populations studies to further confirm our findings.

## Conclusion

The polymorphisms of rs659366 and rs660339 in UCP2 gene and maternal dietary factors were found to be associated with the risk of CHDs in offspring. These results provide the theoretical basis for gene screening and a new clue for the prevention of CHDs in offspring. However, the limitations of our study still should be carefully considered. Whether the study findings can be applied to other groups remains to be explored.

## Materials and methods

### Ethics statement

The study was approved by the Ethics Committee of Xiangya School of Public Health Central South University (No. XYGW-2018-36) and performed according to the Declaration of Helsinki. The design and purpose of the current study were clearly described in the research protocol; the protocol was registered at the Chinese Clinical Trial Registry with registration number ChiCTR1800018492 and is available at http://www.chictr.org.cn/listbycreater.aspx. All participants provided written informed consent before completing an enrollment questionnaire as well as providing biological samples.

### Study design and data sources

We conducted a hospital-based case–control study in Changsha City, Hunan province, China, from March 2018 to August 2019. A total of 803 children with CHD and 889 children without any congenital malformations were recruited in Hunan Children’s Hospital. During recruitment, every mother of children was interviewed face to face by using a structured questionnaire.

A total of 803 mothers who had a fetus with CHDs was recruited to the case group; of these, 124 mothers who were non-Han Chinese, 56 not completing the questionnaire, 26 belonging to multiple pregnancies, and 133 not providing the blood sample were excluded. Finally, a total of 464 cases were involved in our analysis. During the same period, total 889 mothers were recruited in the control group who had a baby without any birth defects; of these, 98 mothers who were non-Han Chinese, 101 not having a complete record of the questionnaire, 19 belonging to multiple pregnancies, and 167 not providing the blood sample were excluded. Not all the selected controls were available, and only 504 controls were included.

### Inclusion criteria

In the present study, the exposures of interest were maternal dietary habits in early pregnancy and the polymorphisms of UCP2 gene. The outcome of interest was CHDs that included the following specific phenotypes: atrial septal defect (ASD), ventricular septal defect (VSD), patent ductus arteriosus (PDA), atrioventricular septal defect (AVSD), tetralogy of Fallot (TOF), and complete transposition of great arteries (TGA).

To minimize recall bias of exposure by mothers to the greatest extent, all cases and controls recruited were younger than 1 year old. All cases were selected from the same hospital but different clinics during the same study period as the cases, which were diagnosed by using ultrasonography and confirmed by surgery. And the control group was recruited from the Department of Child Healthcare after health counseling or medical examination. During recruitment, every mother of children was interviewed face to face by using a structured questionnaire by some professionally trained investigators and provided the blood sample.

### Epidemiological investigation

The field epidemiological investigation mainly included basic demographic criteria gestational age, maternal education level, average annual income, body mass index (BMI), history of diabetes, smoking history, second-hand smoke exposure history, history of drinking alcohol, folic acid supplement, and dietary habits. Among them, a self-administered food frequency questionnaire was developed based on a semi-quantitative food frequency questionnaire (SQFFQ) which have been used to investigate maternal dites during the pregnancy in a previous study^27^ and local eating habits, which was considered validated (The Crohnbach’s α coefficient was 0.81 and test–retest reliability was 0.92). The frequency of consumption was defined as follows: the average weekly intake of food less than twice was defined as no food intake, and the average weekly intake of food more than twice was defined as excessive food intake (i.e., pickled vegetables, smoked foods, barbecued foods, and fried foods) or regular food intake (i.e., fresh meat, fish and shrimp, fresh eggs, fresh vegetables, soy foods, and milk products). After completing the epidemiological investigation, for each mother, three to five milliliters of blood samples were collected in EDTA by venipuncture for genotyping. Plasma and blood cells were separated from the blood by centrifugation and finally stored at − 80 °C for later laboratory analysis.

### SNP selection and genotyping

The genomic DNA was extracted from peripheral blood cells by using the QIAamp DNA Mini Kit (Qiagen, Valencia, CA, USA) based on the manufacturer’s standard protocol and dissolved in sterile TBE buffer. Presently, considering this fact that there few studies on the association between UCP2 gene variants and risk of CHDs in offspring, we selected candidate loci of UCP2 gene mainly based on previous similar studies that assessed the association of UCP2 gene variants with risk of diabetes mellitus and obesity. As a result, these genetic loci including rs659366, rs660339, and rs591758 in UCP2 genes were selected as candidate loci for this study. The polymorphism of UCP2 gene was genotyped using the matrix-assisted laser desorption and ionization time-of-flight mass spectrometry Mass Array system (Agena iPLEXassay, San Diego, CA, USA). The primer sequences of rs659366 were ACGTTGGATGAAACGCACGTGTTTGTCCCG (forward) and ACGTTGGATGTTTAATTGGCTGACCGTCC (reverse). The primer sequences of rs660339 were ACGTTGGATGGATCCAAGGAGAAAGTCAGG (forward) and ACGTTGGATGTGGTCAGAATGGTGCCCATC (reverse). The primer sequences of rs591758 were ACGTTGGATGGGGAAAGCACTGTAAAACCA (forward) and ACGTTGGATGTCTCCCCAACTTCTTAGAGC (reverse). The laboratory technician, who performed the genotyping, retyped and double-checked each sample, and recorded the genotype data, was blinded to whether the samples were from cases or controls. The error rate of genotyping was lower than 5%.

### Statistical analysis

Statistical analysis was performed using SPSS 24.0 software (SPSS Inc., Chicago, IL, USA). Demographic characteristics and the SNP genotypes of UCP2 were evaluated using the Pearson chi-squared test, Fisher’s exact test (for categorical variables), Student’s t-test, and Wilcoxon’s rank-sum test (for continuous variables). Hardy–Weinberg equilibrium (HWE) was tested for every group. Linkage disequilibrium (LD) and haplotype analysis were performed using HaploView 4.2. The associations of UCP2 genetic variants and maternal dietary factors with the risk of CHDs were estimated by calculating odds ratios (ORs) and their 95% confidence intervals (CIs) using logistic regression models. The forest map was performed using SPSS 24.0 software (SPSS Inc., Chicago, IL, USA) and Review Manager Version 5.3 (The Nordic Cochrane Centre, The Cochrane Collaboration). Besides, we also examine additive interactive effects of the UCP2 gene and maternal dietary factors on the risk of developing CHDs in offspring. The relative excess risk due to interaction (RERI), OR, and 95% CIs were calculated using Microsoft Excel according to Knol et al.^48^. The tests were performed significantly for a two-sided *P* value not exceeding 0.05, except where otherwise specified.

## Supplementary Information

Supplementary Information

## References

[1] Triedman JK, Newburger JW (2016). Trends in congenital heart disease the next decade. Circulation.

[2] van der Linde D (2011). Birth prevalence of congenital heart disease worldwide a systematic review and meta-analysis. J. Am. Coll. Cardiol..

[3] Liu Y (2019). Global birth prevalence of congenital heart defects 1970–2017: Updated systematic review and meta-analysis of 260 studies. Int. J. Epidemiol..

[4] Uzark K (2016). Challenges of assessing quality of life in congenital heart disease globally. J. Am. Coll. Cardiol..

[5] Zaidi S, Brueckner M (2017). Genetics and Genomics of Congenital Heart Disease. Circ. Res..

[6] van der Bom T (2011). The changing epidemiology of congenital heart disease. Nat. Rev. Cardiol..

[7] Wang T (2019). Congenital heart disease and risk of cardiovascular disease: A meta-analysis of cohort studies. J. Am. Heart Assoc..

[8] Gaynor JW (2015). Neurodevelopmental outcomes after cardiac surgery in infancy. Pediatrics.

[9] Wang DG (1998). Large-scale identification, mapping, and genotyping of single-nucleotide polymorphisms in the human genome. Science.

[10] Hirschhorn JN, Daly MJ (2005). Genome-wide association studies for common diseases and complex traits. Nat. Rev. Genet..

[11] Palanisamy AP (2014). Mitochondrial uncoupling protein 2 induces cell cycle arrest and necrotic cell death. Metab. Syndr. Relat. Disord..

[12] Li J, Jiang R, Cong X, Zhao Y (2019). UCP2 gene polymorphisms in obesity and diabetes, and the role of UCP2 in cancer. FEBS Lett..

[13] Tian XY, Ma S, Tse G, Wong WT, Huang Y (2018). Uncoupling Protein 2 in Cardiovascular Health and Disease. Front. Physiol..

[14] de Souza BM (2013). Associations between UCP1 -3826A/G, UCP2 -866G/A, Ala55Val and Ins/Del, and UCP3-55C/T polymorphisms and susceptibility to type 2 diabetes mellitus: Case-control study and meta-analysis. PLoS ONE.

[15] Kaabi YA (2018). The deletion polymorphism in Exon 8 of uncoupling protein 2 is associated with severe obesity in a Saudi Arabian case-control study. Indian J. Endocrinol. Metab..

[16] Hu X (2013). Gene polymorphisms of ADIPOQ +45T>G, UCP2 -866G>a, and FABP2 Ala54Thr on the risk of colorectal cancer: A matched case-control study. PLoS ONE.

[17] Chen L (2019). Risk of congenital heart defects in offspring exposed to maternal diabetes mellitus: An updated systematic review and meta-analysis. Arch. Gynecol. Obstet..

[18] Zheng Z (2018). Increased maternal body mass index is associated with congenital heart defects: An updated meta-analysis of observational studies. Int. J. Cardiol..

[19] Zhou TC (2018). Polymorphisms in the uncoupling protein 2 gene are associated with diabetic retinopathy in Han Chinese patients with type 2 diabetes. Genet Test Mol. Biomark..

[20] Gioli-Pereira L (2013). Association between UCP2 A55V polymorphism and risk of cardiovascular events in patients with multi-vessel coronary arterial disease. BMC Med. Genet..

[21] Engineer A, Saiyin T, Greco ER, Feng Q (2019). Say NO to ROS: Their roles in embryonic heart development and pathogenesis of congenital heart defects in maternal diabetes. Antioxidants (Basel)..

[22] Kumar SD, Yong SK, Dheen ST, Bay BH, Tay SS (2008). Cardiac malformations are associated with altered expression of vascular endothelial growth factor and endothelial nitric oxide synthase genes in embryos of diabetic mice. Exp. Biol. Med. (Maywood)..

[23] Botto LD, Correa A (2003). Decreasing the burden of congenital heart anomalies: An epidemiologic evaluation of risk factors and survival. Prog. Pediatr. Cardiol..

[24] Czeizel A, Dudás I, Vereczkey A, Bánhidy F (2013). Folate deficiency and folic acid supplementation: The prevention of neural-tube defects and congenital heart defects. Nutrients.

[25] Smedts HPM (2009). High maternal vitamin E intake by diet or supplements is associated with congenital heart defects in the offspring. Obstet. Gynecol. Surv..

[26] Shaw GM, Carmichael SL, Yang W, Lammer EJ (2010). Periconceptional nutrient intakes and risks of conotruncal heart defects. Birth Defects Res..

[27] Yang J (2019). Maternal dietary patterns during pregnancy and congenital heart defects: A case-control study. Int. J. Environ. Res. Public Health.

[28] Luglio HF, Eurike D, Huriyati E, Julia M, Susilowati R (2016). Gene-lifestyle interaction: The role of SNPs in UCP2 -866G/A and UCP3 -55C/T on dietary intake and physical activity in Indonesian obese female adolescents. Mediterr. J. Nutr. Metab..

[29] Huriyati E (2016). Dyslipidemia, insulin resistance and dietary fat intake in obese and normal weight adolescents: The role of uncoupling protein 2–866G/A gene polymorphism. Int J Mol Epidemiol Genet..

[30] Zhao QM (2014). Pulse oximetry with clinical assessment to screen for congenital heart disease in neonates in China: A prospective study. Lancet.

[31] Shen Y (2014). Investigation of variants in UCP2 in Chinese type 2 diabetes and diabetic retinopathy. PLoS ONE.

[32] Gamboa R (2018). The UCP2 -866G/A, Ala55Val and UCP3 -55C/T polymorphisms are associated with premature coronary artery disease and cardiovascular risk factors in Mexican population. Genet. Mol. Biol..

[33] Chai Y (2012). The uncoupling protein 2–866G > a polymorphism is associated with the risk of ischemic stroke in chinese type 2 diabetic patients. CNS Neurosci. Ther..

[34] Dieter C (2020). 866G/A and Ins/Del polymorphisms in the UCP2 gene and diabetic kidney disease: Case-control study and meta-analysis. Genet. Mol. Biol..

[35] Farias JG (2017). Antioxidant therapeutic strategies for cardiovascular conditions associated with oxidative stress. Nutrients.

[36] Pravednikova AE (2020). Association of uncoupling protein (UCP) gene polymorphisms with cardiometabolic diseases. Mol. Med..

[37] Li J, Jia W, Zhao Q (2014). Excessive nitrite affects zebrafish valvulogenesis through yielding too much NO signaling. PLoS ONE.

[38] Zodl B (2007). Intestinal transport and metabolism of acrylamide. Toxicology.

[39] Jedrychowski W (2012). Impact of barbecued meat consumed in pregnancy on birth outcomes accounting for personal prenatal exposure to airborne polycyclic aromatic hydrocarbons: Birth cohort study in Poland. Nutrition.

[40] Jules GE, Pratap S, Ramesh A, Hood DB (2012). In utero exposure to Benzo(A)pyrene predisposes offspring to cardiovascular dysfunction in later-life. Toxicology.

[41] Huang L (2012). Benzo[a]Pyrene exposure influences the cardiac development and the expression of cardiovascular relative genes in zebrafish (Danio Rerio) embryos. Chemosphere.

[42] Lupo PJ (2012). Maternal occupational exposure to polycyclic aromatic hydrocarbons and congenital heart defects among offspring in the national birth defects prevention study. Birth Defects Res..

[43] Li N (2018). Assessment of interaction between maternal polycyclic aromatic hydrocarbons exposure and genetic polymorphisms on the risk of congenital heart diseases. Sci. Rep..

[44] Zhang BY (2008). Correlation between birth defects and dietary nutrition status in a high incidence area of China. Biomed. Environ. Sci..

[45] Pan XF, Marklund M, Wu JH (2019). Fish consumption for cardiovascular health: Benefits from long-chain omega-3 fatty acids versus potential harms due to mercury. Heart.

[46] Chrysant SG, Chrysant GS (2013). An update on the cardiovascular pleiotropic effects of milk and milk products. J. Clin. Hypertens. (Greenwich)..

[47] Bunin GR, Gyllstrom ME, Brown JE, Kahn EB, Kushi LH (2001). Recall of diet during a past pregnancy. Am. J. Epidemiol..

[48] Knol MJ, VanderWeele TJ (2012). Recommendations for presenting analyses of effect modification and interaction. Int. J. Epidemiol..

