# Supplementary material for: Association and interaction effect of UCP2 gene polymorphisms and dietary factors with congenital heart diseases in Chinese Han population
Source: Sci Rep. 2021 Apr 22;11:8699. doi: 10.1038/s41598-021-88057-2 (PMC8062668; doi:10.1038/s41598-021-88057-2)
Supplement: Supplementary file 1 — Supplementary Information [file 41598_2021_88057_MOESM1_ESM.pdf]

Tille: Association and interaction effect of UCP2 gene polymorphisms and dietary factors with congenital heart diseases in Chinese Han Population

Short title: association and interaction effect of dietary factors and gene on CHDs

Author: Senmao Zhang MPH, Xiaoying Liu MPH, Tingting Wang PhD, Lizhang Chen PhD, Tubao Yang PhD, Peng Huang MD, and Jiabi Qin PhD.

Supplement Table 1. Hardy-Weinberg equilibrium (HWE) test of UCP2 genotype in the control group

Supplement Table 2. Interaction between the recessive model of rs659366 and maternal dietary factors for the risk of CHDs

Supplement Table 3. Interaction between the dominant model of rs660339 and maternal dietary factors for the risk of CHDs

Supplement Table 4. Interaction between the recessive model of rs660339 and maternal dietary factors for the risk of CHDs

Supplement Figure 1. Linkage disequilibrium (LD) analysis of the UCP2 SNPs between cases and controls

Supplement Table 1. Hardy-Weinberg equilibrium (HWE) test of UCP2 genotype in the control group

**Genotype**	**Actual frequency**	**Theoretical frequency**	**Gene frequency**	χ^2	***P***
rs659366				3.16	0.08
CC	252	240.29	0.50		
TC	192	215.43	0.38		
TT	60	48.29	0.12		
rs660339				3.55	0.06
GG	218	208.29	0.43		
GA	212	231.43	0.42		
AA	74	64.29	0.15		
rs591758				2.43	0.12
GG	180	171.50	0.36		
GC	228	245.00	0.45		
CC	96	87.50	0.19		

Supplement Table 2. Interaction between the recessive model of rs659366 and maternal dietary factors for the risk of CHDs

Dietary factors	CC+TC		TT		aOR(95%CI) for genotypes within strata of dietary factors	RERI(95%CI)
	case/control	aOR(95%CI)	case/control	aOR(95%CI)		
**Pickled vegetables**						
Never	206/283	1(Ref.)	47/47	1.34 (0.78-2.31) *P* =0.292	1.34 (0.78-2.31) *P* =0.292	4.29(-0.58-9.15) *P* =0.084
Excessive	162/161	1.56(1.10-2.20) *P* =0.012	49/13	6.18 (2.78-13.76) *P* <0.001	3.97 (1.76-8.93) *P* =0.001	
aORs (95%CI) for excessive pickled vegetables intake within strata of genotype		1.56(1.10-2.20) *P* =0.012		4.61 (1.85-11.49) *P* =0.001		
**Smoked foods**						
Never	149/236	1(Ref.)	41/40	1.69 (0.94-3.01) *P* =0.078	1.69 (0.94-3.01) *P* =0.078	3.15(-0.18-6.47) *P* =0.064
Excessive	219/208	2.19 (1.55-3.11) *P* <0.001	55/20	6.02 (2.95-12.29) *P* <0.001	2.60 (1.36-5.56) *P* =0.005	
aORs (95%CI) for excessive smoked foods intake within strata of genotype		2.19 (1.55-3.11) *P* <0.001		3.57 (1.53-8.33) *P* =0.003		
**Fresh meat**						
Regular	292/404	1(Ref.)	86/58	1.92 (123-3.01) *P* =0.004	1.92 (123-3.01) *P* =0.004	1.19(-4.83-7.21) *P* =0.697
Never	76/40	1.53 (0.95-2.45) *P* =0.081	10/2	3.64 (0.70-18.78) *P* =0.123	2.38 (0.44-12.99) *P* =0.329	
aORs (95%CI) for never fresh meat intake within strata of genotype		1.53 (0.95-2.45) *P* =0.081		1.89 (0.35-10.20) *P* =0.321		

**Fish and shrimp**						
Regular	116/302	1(Ref.)	18/34	1.26 (0.57-2.68)<br>*P* =0.541	1.26 (0.57-2.68)<br>*P* =0.541	1.99 (-1.02-5.02)<br>*P* =0.194
Never	252/142	3.15(2.23-4.47)<br>*P* <0.001	78/26	5.41(3.06-9.57)<br>*P* <0.001	1.72 (0.97-3.03)<br>*P* =0.062	
aORs (95%CI) for never fish and shrimp intake within strata of genotype		3.15(2.23-4.47)<br>*P* <0.001		4.29 (1.79-10.20)<br>*P* =0.001		
**Milk products**						
Regular		1(Ref.)		1.93 (1.13-3.28)<br>*P* =0.016	1.93 (1.13-3.28)<br>*P* =0.016	2.08 (-3.98-8.13)<br>*P* =0.502
Never	174/372	4.47 (3.02-6.62)<br>*P*<0.001	49/50	7 .47 (3.32-16.82)<br>*P* <0.001	1.67 (0.72-3.87)<br>*P* =0.229	
aORs (95%CI) for never milk products intake within strata of genotype	194/72	4.47 (3.02-6.62)<br>*P*<0.001	47/10	3.87 (1.54-9.71)<br>*P* =0.004		

Note: CHDs = congenital heart diseases；aORs = adjusted odds ratios；95%CI = 95% confidence interval; RERI = The relative excess risk due to interaction. Adjusted for baseline characteristics that were significantly different among two groups

Supplement Table 3. Interaction between the dominant model of rs660339 and maternal dietary factors for the risk of CHDs

Dietary factors	GG		GA+AA		aOR(95%CI) for genotypes within strata of dietary factors	RERI(95%CI)
	case/control	aOR(95%CI)	case/control	aOR(95%CI)		
**Pickled vegetables**						
Never	87/143	1(Ref.)	166/187	1.41(0.94-2.12) *P* =0.101	1.41(0.94-2.12) *P* =0.101	0.95(-0.15-2.04) *P* =0.896
Excessive	49/75	1.39(0.81-2.39) *P* =0.235	162/99	2.74(1.76-4.28) *P* <0.001	1.98 (1.16-3.36) *P* =0.012	
aORs (95%CI) for excessive pickled vegetables intake within strata of genotype		1.39(0.81-2.39) *P* =0.235		1.95 (1.31-2.90) *P* =0.001		
**Smoked foods**						
Never	47/122	1(Ref.)	143/154	2.21(1.37-3.56) *P* <0.001	2.21(1.37-3.56) *P* <0.001	0.01(-1.79-1.81) *P* =0.994
Excessive	89/96	3.13(1.83-5.34) *P* <0.001	185/132	4.35(2.66-7.11) *P* <0.001	1.39 (0.88-2.18) *P* =0.153	
aORs (95%CI) for excessive smoked foods intake within strata of genotype		3.13(1.83-5.34) *P* <0.001		1.96 (1.32-2.93) *P* =0.001		
**Fresh meat**						
Regular	112/208	1(Ref.)	286/274	1.69 (1.21-2.37) *P* =0.002	1.69 (1.21-2.37) *P* =0.002	0.77 (-2.39-3.93) *P* =0.632
Never	24/10	2.13 (0.90-5.02) *P* =0.086	42/12	3.59 (1.65-7.80) *P* =0.001	1.69 (0.57-5.05) *P* =0.348	
aORs (95%CI) for never fresh meat intake within strata of genotype		2.13 (0.90-5.02) *P* =0.086		2.12 (1.00-4.50) *P* =0.051		

**Fish and shrimp**						
Regular	102/208	1(Ref.)	264/274	1.77 (1.25-2.49)<br>*P* =0.569	1.77 (1.25-2.49)<br>*P* =0.569	
Never	34/10	4.62 (1.91-11.18)<br>*P* =0.001	64/12	5.02 (2.46-10.21)<br>*P* <0.001	1.09(0.37-3.15)<br>*P* =0.880	-0.37<br>(-5.49-4.76)<br>*P* =0.889
aORs (95%CI) for never fish and shrimp intake within strata of genotype		4.62 (1.91-11.18)<br>*P* =0.001		2.84 (1.43-5.65)<br>*P* =0.003		
**Milk products**						
Regular	68/181	1(Ref.)	155/241	1.52 (1.02-2.28)<br>*P* =0.041	1.52 (1.02-2.28)<br>*P* =0.041	
Never	68/37	3.80 (2.13-6.79)<br>*P* <0.001	173/45	7.23 (4.37-11.98)<br>*P* <0.001	1.90 (1.04-3.47)<br>*P* =0.037	2.91 (-0.45-6.30)<br>*P* =0.089
aORs (95%CI) for never milk products intake within strata of genotype		3.80 (2.13-6.79)<br>*P* <0.001		4.76 (3.00-7.52)<br>*P* <0.001		

Note: CHDs = congenital heart diseases；aORs = adjusted odds ratios；95%CI = 95% confidence intervals; RERI = The relative excess risk due to interaction. Adjusted for baseline characteristics that were significantly different among two groups

Supplement Table 4. Interaction between the recessive model of rs660339 and maternal dietary factors for the risk of CHDs

Dietary factors	GG+ GA		AA		aOR(95%CI) for genotypes within strata of dietary factors	RERI(95%CI)
	case/control	aOR(95%CI)	case/control	aOR(95%CI)		
**Pickled vegetables**						
Never	195/275	1(Ref.)	58/55	1.42(0.87-2.32)<br>*P* =0.166	1.42(0.87-2.32)<br>*P* =0.166	3.28(-0.42-6.99)<br>*P* =0.082
Excessive	157/155	1.58(1.11-2.25)<br>*P* =0.011	54/19	5.28(2.58-10.81)<br>*P* <0.001	3.34 (1.61-6.94)<br>*P* =0.001	
aORs (95%CI) for excessive pickled vegetables intake within strata of genotype		1.58(1.11-2.25)<br>*P* =0.011		3.73(1.66-8.33)<br>*P* =0.001		
**Smoked foods**						
Never	136/230	1(Ref.)	54/46	1.88(1.10-3.20)<br>*P* =0.021	1.88(1.10-3.20)<br>*P* =0.021	1.86(-1.25-4.96)<br>*P* =0.242
Excessive	216/200	2.31(1.62-3.30)<br>*P* <0.001	58/28	5.05(2.67-9.54)<br>*P* <0.001	2.18 (1.17-4.07)<br>*P* =0.014	
aORs (95%CI) for excessive smoked foods intake within strata of genotype		2.31(1.62-3.30)<br>*P* <0.001		2.69 (1.27-5.68)<br>*P* =0.010		
**Fresh meat**						
Regular	296/409	1(Ref.)	102/73	1.84 (1.23-2.76)<br>*P* =0.003	1.84 (1.23-2.76)<br>*P* =0.003	4.49 (-11.37-20.34)<br>*P* =0.579
Never	56/21	2.06 (1.14-3.72)<br>*P* =0.017	10/1	7.39 (0.87-63.09)<br>*P* =0.068	1.84 (1.23-2.76)<br>*P* =0.257	
aORs (95%CI) for never fresh meat intake within strata of genotype		2.06 (1.14-3.72)<br>*P* =0.017		4.02 (0.46-34.48)<br>*P* =0.209		

**Fish and shrimp**						
Regular	282/416	1(Ref.)	84/66	2.01 (1.31-3.10)<br>*P* =0.002	2.01 (1.31-3.10)<br>*P* =0.002	-3.22 (-7.16-0.71)<br>*P* =0.108
Never	70/14	4.74 (2.41-9.32)<br>*P* <0.001	28/8	2.53 (1.06-6.04)<br>*P* =0.037	0.53 (0.18-1.56)<br>*P* =0.251	
aORs (95%CI) for never fish and shrimp intake within strata of genotype		4.74 (2.41-9.32)<br>*P* <0.001		1.26 (0.49-3.22)<br>*P* =0.634		
**Milk products**						
Regular	176/356	1(Ref.)	47/66	1.51 (0.92-2.47)<br>*P* =0.102	1.51 (0.92-2.47)<br>*P* =0.102	7.11 (-2.57-16.79)<br>*P* =0.150
Never	176/74	3.96 (2.67-5.87)<br>*P* <0.001	65/8	11.57 (4.95-27.08)<br>*P* <0.001	2.92 (1.22-6.99)<br>*P* =0.016	
aORs (95%CI) for never milk products intake within strata of genotype		3.96 (2.67-5.87)<br>*P* <0.001		7.69 (4.95-27.03)<br>*P* <0.001		

Note: CHDs = congenital heart diseases; aORs = adjusted odds ratios; 95%CI = 95% confidence intervals; RERI = The relative excess risk due to interaction. Adjusted for baseline characteristics that were significantly different among two groups

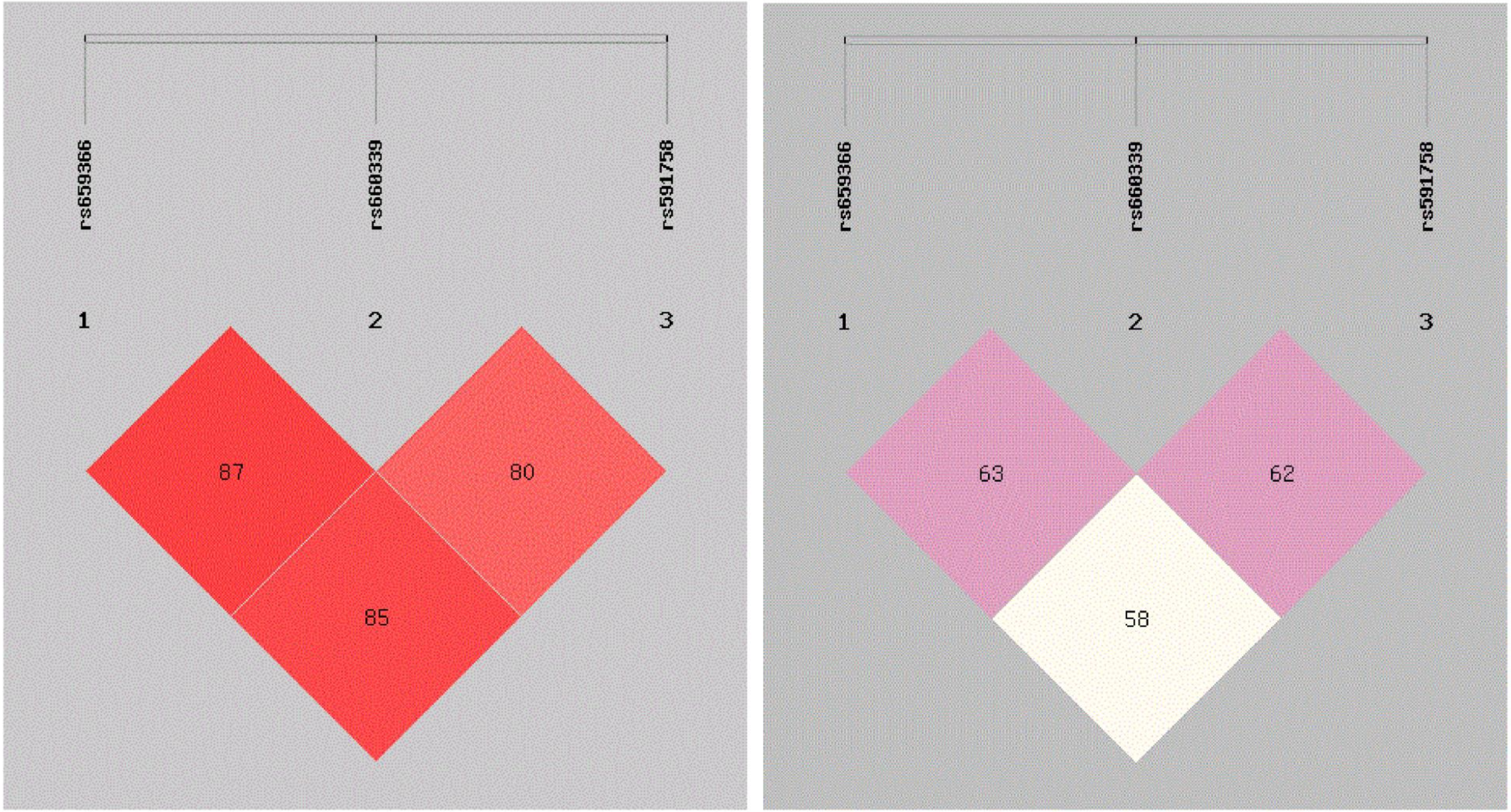

Supplement Figure 1. Linkage disequilibrium (LD) analysis of the UCP2 SNPs between cases and controls
